# Through a Tainted Lens: A Qualitatve Study of Medical Learners’ Thinking About Patient ‘Deservingness’ of Health Advocacy

**DOI:** 10.5334/pme.1314

**Published:** 2024-02-23

**Authors:** Ian Scott, Maria Hubinette, Theresa van der Goes, Renate Kahlke

**Affiliations:** 1 Department of Family Practice and the Director of the Centre for Health Education Scholarship at the University of British Columbia, Vancouver British Columbia, Canada; 2 Department of Family Practice and a Scholar at the Centre for Health Education Scholarship at the University of British Columbia, Vancouver BC, Canada; 3 Department of Family Practice, University of British Columbia, Vancouver BC, Canada; 4 Division of Education and Innovation, Department of Medicine and Scientist in the McMaster Education Research, Innovation and Theory Program at McMaster University, Hamilton, Ontario, Canada

## Abstract

**Introduction:**

While health advocacy is a key component of many competency frameworks, mounting evidence suggests that learners do not see it as core to their learning and future practice. When learners do advocate for their patients, they characterize this work as ‘going above and beyond’ for a select few patients. When they think about advocacy in this way, learners choose who deserves their efforts. For educators and policymakers to support learners in making these decisions thoughtfully and ethically, we must first understand how they are currently thinking about patient deservingness.

**Methods:**

We conducted qualitative interviews with 29 undergraduate and postgraduate medical learners, across multiple sites and disciplines, to discuss their experiences of and decision-making about health advocacy. We then carried out a thematic analysis to understand how learners decided when and for whom to advocate. Stemming from initial inductive coding, we then developed a deductive coding framework, based in existing theory conceptualizing ‘deservingness.’

**Results:**

Learners saw their patients as deserving of advocacy if they believed that the patient: was not responsible for their condition, was more in need of support than others, had a positive attitude, was working to improve their health, and shared similarities to the learner. Learners noted the tensions inherent in, and discomfort with, their own thinking about patient deservingness.

**Discussion:**

Learners’ decisions about advocacy deservingness are rooted in their preconceptions about the patient. Explicit curriculum and conversations about advocacy decisions are needed to support learners in making advocacy decisions equitably.

## Introduction

Advocacy work has been promoted as a means to reduce health disparities and, as such, doctors and medical learners in many countries are required to engage in health advocacy for patients and populations [1,2,3,4,5] or ‘contribute their expertise and influence as they work with communities or patient populations to improve health’ [6,7]. However, much of learners’ advocacy activities focus on individual patients [8]. Unfortunately, the skills required to do individual patient advocacy successfully have proven difficult for learners to acquire. Health advocacy receives little attention in curriculum and assessment compared to other aspects of medical education [9] and many feel unprepared to do advocacy work when they graduate [9,10].

When learners do advocate, they often characterize these activities as going ‘above and beyond’ their core medical duties for only a few of their patients [8]. At first glance, ‘going above and beyond’ for some patients appears admirable – an effort to help those who need it most. However, it is not clear how learners are making decisions about which patients will receive their advocacy efforts when they choose to go above and beyond. And while triaging patients’ relative need of healthcare services is a core component of physicians’ work in overburdened systems, these providers are also human and bring their assumptions and biases to the table when making these decisions [11,12]. Given that advocacy is so under-taught, learners are likely to receive little or no support to guide them in their decisions about when and for whom to advocate. As a result, there is reason to believe that advocacy efforts are not always applied equitably [13,14,15,16,17,18]. In order to support learners in bringing intention to their advocacy efforts, we need to better understand how they are currently making advocacy decisions.

The construct of deservingness has been explored extensively in social policy research as a way of understanding citizens’ perspectives on where – and for whom – social benefits, such as income support, should be deployed [19,20]. This conceptualization of deservingness [19] offers theoretical support for understanding the kinds of valuations and assumptions learners make when choosing which patients will receive their advocacy efforts. Using deservingness theory to guide our analysis, we asked: how do learners make decisions about when and for whom to advocate? Until we are able to unpack learners’ thinking about who deserves their advocacy, we cannot hope to support them in distributing their advocacy resources with intention and equity.

## Methods

We used a basic constructivist qualitative approach to explore learners’ thinking about deservingness. Basic qualitative studies offer a flexible approach that can be responsive to new findings [21], given that researchers build their methodologies to fit their evolving questions. In keeping with this approach, we ‘borrowed’ elements of constructivist grounded theory (CGT) [22] early in the study to inform sampling, data generation and initial inductive analysis [23]. Later thematic analyses were deductive, allowing us to capitalize on the explanatory power of existing theory [24], as described below.

### Sampling and Recruitment

This study took place at a large Canadian medical school with training sites distributed across the province. Recruitment occurred as part of a large CGT study exploring learner interpretations of health advocacy [8]. We recruited medical students and family medicine residents by email and in-classroom announcements. Initial recruitment included 48 residents and 80 medical students across diverse rural, semi-rural, and urban sites who consented to allow us to view their assessments (family medicine residents: field notes related to advocacy; medical students: reflections focussed on diversity and equity) and to contact them for an interview. These documents were used to select an initial purposeful sample (N = 16) for interviews, based on diversity in their perspectives on health advocacy (assessed through their advocacy-related assessments), geographic location, and patient populations served.

Our initial analysis of seven interviews identified ‘patient deservingness’ as central to learners’ decisions about advocacy. To capture this important concept, we modified the interview guide (see Appendix). As part of the main study, we also employed theoretical sampling to include learners who were likely to hold divergent perspectives. Specifically, we recruited medical students and residents who appeared to have a less developed understanding of advocacy (based on their assessments), residents outside of family medicine (paediatric and internal medicine), residents working in remote locations, and residents from an Indigenous-focused residency program (N = 13). In total we recruited 29 participants.

### Data Generation

Participants each completed 60 minutes individual semi-structured interview [25] in-person, online via Skype, or via telephone. Four Internal medicine residents participated in one group interview of about 1.5 hours, in order to explore the differing interpretations and experiences of advocacy (including group consensus and dissensus), within this generalist but primarily hospital-based speciality. In an effort to ensure participants’ comfort and anonymity, all individual interviews were conducted by team members who were at arms-length from their education and assessment (RK for individual, MH for group interviews). Table 1 offers details on participant demographics.

**Table 1:** Participant Demographics.


**Level of Training**	Undergraduate 9 Postgraduate 20

**Specialty (Postgraduate)**	Family Medicine 14 Paediatric Medicine 2 Internal Medicine 4 Undergraduate 9

**Geographic Location**	Location A 3 Location B 2 Location C 20 Location D 4

**Practice Settings** (Some participants were learning in more than one setting or with multiple populations, so may be counted in multiple categories)	Urban or Suburban 28 Rural or semi-rural 9 Remote 1

**Gender**	Women 13 Men 16


In early interviews learners spoke spontaneously about deservingness as a factor that guided advocacy decisions, so we adjusted the interview guide to generate additional data about how they decided to advocate, (e.g. ‘Are there some patients that you advocate for more often? Less often?’ See appendix for interview guide). Data generation continued until the study team felt that they had achieved theoretical sufficiency, and new data or analysis did not yield new insights [26].

### Data Analysis and Theoretical Framework

We managed and analyzed data using Nvivo software (Version 11/12). During initial coding, three team members (RK, MH and a research assistant (RA)) coded all transcripts, staying close to the data and attempting to avoid early abstraction. The other team members independently coded a subset of ten transcripts selected for diversity. The team then met to develop a provisional focused coding framework. In these discussions, we were struck by learners’ discussions of patient deservingness. Consistent with CGT, we continued to explore the literature related to our data as we engaged in data generation and analysis [23]. As we progressed through inductive analysis, we identified an existing theory of deservingness that offered insight into how participants were thinking about patient deservingness.

The deservingness framework, from social policy research, includes five domains, referred to by the acronym CAIRN: control, attitude, identity, reciprocity, and need [19]. Control relates to the perceived degree of *control* that citizens felt a potential recipient of social assistance has over their situation—were they seen as responsible for the challenges they faced? Citizens also made judgements about the potential recipient’s perceived *attitude*. Recipients were seen as more deserving if they seemed grateful, for example. A perception of shared *identity* was also key to deservingness—when citizens perceived similarities between themselves and the potential recipient, the recipient was seen as more deserving. *Reciprocity*, or the perceived expectation that the recipient will ‘pay back’ the benefits in some way (e.g., by paying many years of taxes), also factored into deservingness. Lastly, citizens made deservingness decisions based on the extent to which they felt the potential recipient was truly in *need* of support. For our purposes, CAIRN offered theoretical support from well-established theory [24], deepening our analysis.

When assessing the ‘fit’ of CAIRN to our data, we compared our initial inductive codes to the CAIRN domain descriptions, finding close overlap. We then developed a new coding framework, based on CAIRN [19] and IS recoded the data deductively [27]. The other authors (MH, RK, TVG) then deductively coded a subset (5 transcripts) for discussion.

### Reflexivity and Rigour

The overall study was managed and led by RK whose expertise in qualitative research and sociocultural perspectives informed much or our approach. MH is a family physician with significant experience in curriculum development and medical education research on health advocacy. She helped to situate our findings in the advocacy literature and educational practice. Both IS and TvdG are family physicians and medical educators who used experience in program administration (IS) and assessment (TvdG) to contextualize findings. None of the authors had considered the concept of deservingness as a driver of advocacy when we began this study. However, our perspectives did help us think about advocacy as part of a larger social/practice context. To capitalize on our team’s diversity and ensure that all team members’ perspectives were grounded in the data, each of us handled data during each stage of analysis.

Given that some groups of learners within this study were very small and might be easily identified, we have deidentified data and labeled quotes from learners as PMR (paediatric medicine resident), FMR (family medicine resident), IMR (internal medicine resident) and UGM (undergraduate medical student).

This research was approved by the University of British Columbia’s Research Ethics Board (H16-01818).

## Results

Learners rarely spoke about advocacy at a group or population level and primarily focused on individual advocacy for (not with) a patient. When we asked learners how they made decisions about when and for whom to advocate, their responses aligned closely with domains of the CAIRN framework. Learners reported that they considered patient **need**, cooperativeness **(attitude)**, effort toward their own care **(reciprocity)**, and **control** over, or blame for, their current condition or situation when determining the patient’s deservingness of their advocacy efforts. Finally, learners noted that the degree to which they identified **(identity)** with their patient also influenced their choice to advocate.

We found that the elements of the CAIRN framework were not always discrete – they often overlapped or interacted, and were at times in tension with each other. The results sections below map onto the CAIRN domains, though we have adjusted the order to enhance flow and readability as well as highlight tensions between domains.

### Need

Given their context of limited time and resources, most learners in our study discussed the necessity of triaging patients’ need for advocacy, expressing that they were: ‘more likely to advocate for people that … are more in need’ (UGM1). Patients that were seen as having minor problems—whether or not others would perceive those same complaints in the same way—were assessed as less in need and therefore less deserving of advocacy. For example, some learners noted that patients who suffered from certain mental health issues were less likely to receive advocacy:

When patients come in with some kind of psychosomatic or ‘soft’ psych issues, so some kind of anxiety, pseudo-depression, whether it’s just not feeling well, … it’s just like this isn’t something that’s going to kill them.… There’s still some amount of advocacy with that patient’s overall being that is left by the wayside. (UGM2)

In another example, patients from more affluent backgrounds were seen as less deserving because their needs didn’t include the financial and social complexities experienced by less privileged patients. When asked if there were any patient populations for whom they struggle to advocate, one resident related that they are less likely to advocate for patients from affluent backgrounds ‘because I kind of feel like, oh man, like, this is a problem for you? Really? You’ve got everything’ (PMR1).

We note that learner beliefs about patient needs were most often based on initial perceptions, and rarely included other perspectives– such as the patient’s perspective on their own need.

### Attitude (Demandingness/Cooperativeness)

Many learners described advocating more for patients that were cooperative. Conversely, several learners noted that ‘patients that are kind of abrasive and oppositional don’t make me feel like I really want to advocate as strongly for them’ (IMR1). These patients were seen as ‘difficult’, and thus less likely to receive advocacy: ‘when [patients are] not cooperative, it’s really hard on the staff. It’s really hard on everybody. And, it’s difficult for [us] to not look at [these patients] through a tainted lens’ (UGM3). In other words, when learners and staff make decisions about how to allocate their advocacy resources, they often do so through ‘a tainted lens’ – advocacy is conditional, and patients who are uncooperative are seen as undeserving.

However, we note that elements of deservingness interacted with each other in a complex manner. For example, when a patient was perceived as ‘demanding’ (*attitude*), a learner might question their assessment of the patient’s *need*, noting that patients:

May need more advocacy but they’re sort of demanding and I guess the histrionic aspect of them makes it less appealing to do all those things [advocacy activities] and it’s hard – and then it becomes a fine line, like do they actually need this or are they abusing the system and asking for more than they do need. So, I find those patients are quite tough. (IMR2)

### Effort/Reciprocity

Learners also reported advocating more often when they felt that patients were making efforts toward improving their own health. These patients were seen as reciprocating the learner’s advocacy efforts – there was a perception that both parties were working together to improve the patient’s health. One learner noted that patients were easier to advocate for if they were:

Really willing to kind of tackle things. People that are kind of empowered about their own health too and they just need help kind of connecting the dots or people that are just willing to make changes and they’re easier to work with really. (IMR1).

Conversely, learners articulated that they were less likely to advocate when patients did not make reciprocal efforts. One learner stated that when patients complain but ‘don’t do anything about it [their health issues] … those people frustrate me … and I think it does affect how easy or hard it is for me to advocate for those patients’ (IMR3). Put succinctly, learners felt that: ‘it’s my role to work hard for patients but there needs to be some form of effort on the patient’s side as well’ (IMR3).

However, active patients could also be seen as demanding and having a poor attitude when, for example, ‘they’re asking for letters off of work and they’re asking for this paperwork to get filled out and they’re asking for referrals to these various people and they may *need* more advocacy but they’re sort of demanding’ (IMR2). Thus, a patient could be perceived as too demanding when they advocate for themselves, yet if they are perceived as passive or undemanding, they may be seen as unwilling to reciprocate the learner’s efforts. These results show the complexity of how learners perceive effort/reciprocity by patients and how those perceptions influence learners’ decisions about whether or not they will choose to advocate.

### Blame, Control and Responsibility

Many learners reported that patients who were seen as responsible for their health situation were less deserving of advocacy. However, no learner said that they currently felt this way about patients, but did identify others that were guilty of blaming patients for their condition (or that they, themselves had engaged in this blame in the past). For example, one learner stated:

I think it makes it easier for *some people* [emphasis added] to just say ‘well this person’s an addict and they chose this lifestyle therefore they deserve to continue in this lifestyle.’ Whereas…I think a lot of these issues are centred around addictions and housing and I think my view of it as a medical problem makes it easier for me to say this patient might benefit from this or that [form of advocacy] (IMR4).

Another learner shared that while they used to think that addiction was within the control of the patient, they came to realize that addiction was not the patient’s fault: ‘Prior to medical school, I viewed this group of people as suffering more from faults in their own character. I now believe the ‘it’s your fault’ view of drug addiction is a broadly held misconception.’ (UGM2). We believe it is notable that culpability for illness was named by many learners when they were speaking about which patients were deserving of advocacy efforts—they simply never disclosed, or perhaps recognized, that this was currently a means by which they made advocacy decisions.

### Identification and Acquired Empathy

Several learners noted that when they perceived common identities with the patient (e.g., age, gender, religion, country of origin), they often felt an immediate connection and were more likely to advocate. One learner disclosed that ‘*[advocacy] is easier when I feel like I’m personally similar to the patient*’. (UGM3). Another learner highlighted the important role of identification and connection: ‘the biggest driver [of advocacy] is feeling connected with your patient, connected with the family’. (PMR1)

However, many of these learners also noted that their initial impressions of patients were not fixed but could be moderated through meaningful interaction. For example, one learner stated that their empathy grew when they invested time connecting with their patient—even if they initially perceived a patient as very different from them. Thus, they were more likely to engage in advocacy when they:

Learn more about the person. There’s situations you don’t personally understand well, you can better understand it. And I know I felt that way with some of the addictions population because that’s an area of medicine – that’s not something I’ve had a lot of experience before. So, I … just spent extra time talking to them about their lives to kind of figure out where they’re coming from. (UGM4)

Thus, it appears that learners can develop empathy for and understanding of a patient despite a lack of initial perceived similarity with their patient, and this growing empathy appeared to result in increased advocacy.

### Learners’ Efforts to Improve Advocacy Decision Making

Some learners recognized the problematic nature of their advocacy decisions and expressed discomfort when they disclosed their own biases, prefacing their comments with phrases such as ‘as bad as it sounds’ (UGM4) or ‘it doesn’t make you feel as good knowing that you’re leaving some people behind’ (IMR1). These qualifying phrases expressed a tension between learners’ current decision-making practices and their desire to provide equitable care. At the same time when learners recognized the gap between their current and desired practices, they also expressed a desire ‘to attempt to become aware of some of my own biases in order to avoid providing different levels of care to patients who represent diversity in my patient population’ (UGM5). With some seeking to develop ‘specific approaches. I guess it would be nice to have some consistency in terms of the things that you really advocate for’ (IMR1) that could include focusing on a specific area ‘to kind of have a couple of things that you really do push patients to consider and it doesn’t make it feel like overwhelming’ (IMR1) to the learner.

## Discussion

We found learners invoked judgments about patient deservingness that aligned with domains from a published deservingness framework (CAIRN) when making decisions about when and for whom to advocate. We stress that learners’ perceptions of the patient were derived through each learner’s unique perspective – and attendant biases – and they likely do not accurately reflect the patient’s reality. As we note above, learners were sometimes aware that their decisions were filtered through ‘a tainted lens.’ Some expressed both an awareness of and discomfort with their advocacy decision-making practices. Some learners mentioned a desire to address their biases or develop strategies to make their advocacy decision-making more equitable.

Unfortunately, this awareness of, and discomfort with, self-identified bias may not necessarily lead to better advocacy actions by the learner. While Sukhera et al. identified a promising acceptability among providers to address their own biases about their patients [28], it can be extremely difficult for individuals to identify and address biases [29,30,31,32]. By definition, our own biases are always, to some extent, unknown to us [30]. Moreover, seeking to address implicit bias in healthcare professionals raises a sticking point– drawing attention to a provider’s bias can create identity dissonance and lead to the dismissing of such feedback [28]. In our study some participants exhibited awareness of potential biases, but very few identified concrete plans for addressing these biases. Thus, learner openness to thinking about bias is not a sufficient remedy to these challenges and greater curricular time and attention may be needed to move beyond awareness.

The CAIRN deservingness framework may support learners in moving beyond self identification of bias, offering a structure for educator-facilitated reflection on their advocacy decisions. Using tools like CAIRN to support critical reflection within a community can create structure and accountability that supports change—change that would be unlikely when individuals reflect in isolation [33]. For example, learners within a community could be encouraged to critically reflect and discuss with others how their perceptions of patient control, attitude, identity, reciprocity, and need is impacting their advocacy decisions. Faculty development will be required to ensure that educators are prepared to effectively guide conversations that may trigger discomfort and identity dissonance among learners (and teachers). See Table 2 for additional examples.

**Table 2:** Examples of Teaching Interventions to Support Learners’ Advocacy Decision-Making.


CONTEXT/SETTING	EXAMPLES

School/program	Explore how deservingness and resulting bias may influence both curriculum leaders and the curriculum choices they are making for their students (i.e., patient populations, Case Based Learning/Problem Based Learning (CBL/PBL) narratives and how the institution conceives of advocacy in all aspects of their program)

Admissions	Use CAIRN framework to create admissions scenarios (i.e., multiple medical interview—MMI)

Classroom Teaching	Session exploring the impacts of application of a deservingness heuristic on patients

	Critically reflect on a patient case (simulated or real) using the CAIRN framework

Clinical Teaching	Trainees carry out a CAIRN assessment of a patient with input from peers and other providers—do others see the patient the same way? Why or why not?

	Have trainees fill out a CEX on a patient that they have just met and a patient they have a longer-term relationship with to explore the concept of identity in deservingness assessments in order to help learners appreciate how patient engagement/understanding can support their understanding of patient need

Teaching Sites	Have students and/or preceptors carry out a CAIRN critical review of the site (i.e., what is the perception of how the people in that site generally view patients)


Beyond this focus on how individuals assess deservingness, educators may wish to explore how deservingness is baked into systems that impact learner advocacy decisions. Examining the impact of explicit and implicit curricula in driving ‘deservingness’ narratives will be critical if we are to support learners and teachers in unpacking these issues. Out of this work, educators may be able to identify curricular activities that support learners in gaining a deeper understanding of how their thinking about deservingness impacts patient care (see Table 2). This work will not be easy, given the complexities and interactions we have highlighted in learners’ thinking about the deservingness domains. But if we do not address the role of ‘deservingness’ in learners’ distribution of their advocacy, educators will miss a valuable opportunity to support learners in resolving their discomfort with the ‘tainted lens’ through which they currently make advocacy decisions. If we fail to support learners in making nuanced advocacy decisions, we will ultimately fail to support patients who are currently left without much-needed advocacy resources.

## Limitations and Future Directions

We sought to include diverse learners and practice contexts, using theoretical sampling to seek out novel perspectives. Despite our efforts to recruit learners who held diverse views and operated in diverse contexts, those who agreed to participate in this unremunerated study may share perspectives that are not held by a broader population. Our field would benefit from interprofessional, multi-specialty, multi-centre, and international studies that explore the extent to which these conceptions of deservingness are culturally-bound.

## Conclusions

Although health advocacy is an expected component of medical practice, in our under-resourced healthcare systems learners must make tough decisions about when and for whom to advocate. We found that learners consider the ‘deservingness’ of their patient, including their perceptions of the patient’s need, attitude, effort toward their own health (reciprocity), blame for or control over their health problems, and similarity to themselves when deciding to advocate. Many of the assumptions that learners base these decisions upon may be rooted in the learner’s bias and not the reality of the patient.

## Additional File

The additional file for this article can be found as follows:

10.5334/pme.1314.s1 Appendix. Advocacy in Context Interview Guide.
