# Supplementary material for: Through a Tainted Lens: A Qualitatve Study of Medical Learners’ Thinking About Patient ‘Deservingness’ of Health Advocacy
Source: Perspect Med Educ. 2024 Feb 23;13(1):151–9. doi: 10.5334/pme.1314 (PMC10885826; doi:10.5334/pme.1314)
Supplement: Appendix. — Advocacy in Context Interview Guide. [file pme-13-1-1314-s1.pdf]

Advocacy in Context Interview Guide

- **Tell me about a time that you acted as an advocate**
  - **What went into your decision to act?**
  - **How do you decide when to act, given the limits on time and resources?**

- **Are there some patients that you advocate for more often?**
  - **Did you encounter any barriers to acting?**
  - **Were there any consequences to acting?**
    - **Benefits?**

- **Tell me about a time that you wish you had advocated but didn't**
  - **What prevented you from acting?**
  - **How do you feel about your decision?**
  - **Is there anything you did do to mediate the situation?**
  - **Are there any other times that you can tell me about?**

- **What do you think are the main things that make people want to advocate?**

- **What do you think are the main things that support that advocacy?**
  - **For students?**
  - **For residents?**
  - **For professionals?**
